# Identification of BAG5 as a Potential Biomarker for Parkinson’s Disease Patients With R492X *PINK1* Mutation

**DOI:** 10.3389/fnins.2022.903958

**Published:** 2022-07-27

**Authors:** Yu Fu, Yongkang Chen, Haiyan Tian, Han Liu, Dan Qi, Erxi Wu, Xuejing Wang

**Affiliations:** ^1^Department of Neurology, The First Affiliated Hospital of Zhengzhou University, Zhengzhou, China; ^2^Institute of Parkinson’s Disease and Movement Disorders, Zhengzhou University, Zhengzhou, China; ^3^Neuroscience Institute and Department of Neurosurgery, Baylor Scott & White Health, Temple, TX, United States; ^4^Department of Surgery, Texas A&M University Health Science Center College of Medicine, Temple, TX, United States; ^5^Department of Pharmaceutical Sciences, Texas A&M University Health Science Center, College of Pharmacy, College Station, TX, United States; ^6^Livestrong Cancer Institutes, Dell Medical School, The University of Texas at Austin, Austin, TX, United States

**Keywords:** R492X *PINK1* mutation degradation, Bcl2-associated athanogene 5, ubiquitination, Parkinson’s disease, skin

## Abstract

Parkinson’s disease (PD) is a degenerative, progressive nervous system disorder with an unknown cause. *PINK1* [phosphatase and tensin homolog deleted on chromosome 10 (PTEN)-induced putative kinase 1] causative mutations R492X may cause autosomal recessive early-onset parkinsonism. In this study, we utilized patient samples and cell line system to investigate roles of Bcl2-associated athanogene 5 (BAG5) in PD patients with R492X *PINK1* mutation. We show that the expression levels of BAG5 in the skin tissues from PD patients with R492X *PINK1* mutation are markedly lower than those from the healthy control subjects in a small cohort of patients, which has not been recognized before. In addition, we demonstrate that BAG5 physically binds to R492X mutated PINK1 protein. Furthermore, we reveal that BAG5 promotes the degradation of R492X mutated PINK1 protein via ubiquitin/proteasome-dependent pathway, suggesting that decreased level of BAG5 may lead to R492X mutated PINK1 protein accumulation. These findings suggest that BAG5 may serve as an early detection biomarker for PD patients with R492X *PINK1* mutation and provide important new insights on how BAG5 affects R492X mutated PINK1 protein, highlighting therapeutic targets for this disease.

## Introduction

The phosphatase and tensin homologue deleted on chromosome 10 (PTEN)-induced putative kinase 1 (*PINK1*) is a mitochondria-targeted serine/threonine kinase with a mitochondrial localization signal (MLS) domain and a functional serine/threonine kinase domain (Matsuda et al., 2013; Wang et al., 2014; Vizziello et al., 2021; Kakade et al., 2022). MLS parts include a mitochondrial-targeting sequence (MTS) and a putative transmembrane domain (TD), the degradation of N-terminal domain was reported to affect the cellular location of *PINK1* (Eldeeb and Ragheb, 2020; Sekine, 2020; Vizziello et al., 2021). *PINK1* has been well-known for the notion of neuroprotective roles, as it protects cells from damage-mediated mitochondrial dysfunction (Brunelli et al., 2020). In addition, *PINK1* is also known to be implicated in various processes like apoptosis and mitophagy (Voigt et al., 2016; Li et al., 2022). The *PINK1* (or *PARK6*) gene was identified to be linked to Parkinson’s disease (PD) for the first time by linkage analysis of consanguineous families with early-onset autosomal recessive PD and is recently reported as one of the most commonly mutated genes in early onset PD (Valente et al., 2004; Agarwal and Muqit, 2022; Gan et al., 2022). More than 50 *PINK1* mutations have been found to impair mitochondrial functional throughout the kinase and carboxyl-terminal regulatory domains of *PINK1* (Rochet et al., 2012; Matsuda et al., 2013). The R492X mutation in the *PINK1* gene was identified by Hatano et al. (2004). Despite the neurotoxicity of R492X mutated PINK1 protein, inducing mitochondrial dysfunction and oxidative stress, the underlying molecular mechanisms are still to be explored (Leites and Morais, 2021).

The Bcl-2 associated athanogene (BAG) family is a group of chaperone regulators (Qin et al., 2016). All members of the BAG protein family contain BAG domain (BD), which mediates direct interaction with the ATPase domain of Hsp70/Hsc70 molecular chaperones (Bracher and Verghese, 2015). The BAG family proteins perform diverse functions including apoptosis and protein degradation (Bracher and Verghese, 2015). BAG5 is a unique member of the BAG family with five BDs. Previous studies have reported that BAG5 inhibited both Parkin E3 ligase and Hsp70 chaperone activities thereby enhancing dopaminergic neuron degeneration (Berliner et al., 1986; De Snoo et al., 2019). This suggests that BAG5 is involved in proteasome-mediated protein degradation, which is also associated with Parkinson’s disease. In addition, two early studies demonstrated that BAG5 inhibited MPP^+^-induced apoptosis through both endogenous and mitochondria-mediated pathways of apoptosis (Peviani et al., 2012; Wang et al., 2014).

In this study, we first found that the expression levels of BAG5 in the skin tissues from a small cohort of PD patients with R492X *PINK1* mutation markedly decreased compared with those from the healthy control subjects. Furthermore, we demonstrated that BAG5 promotes the degradation of R492X mutated PINK1 protein via ubiquitylation-dependent pathway. These data suggest that BAG5 may serve as an early detection biomarker for PD patients with R492X *PINK1* mutation and provide important new insights on how BAG5 affects R492X mutated PINK1 protein, highlighting therapeutic targets for this disease.

## Materials and Methods

### Parkinson’s Disease Patients and Skin Biopsy

Skin tissues were obtained from two patients with PD harboring R492X *PINK1* mutation and two healthy controls. Punch biopsy of the skin (3 mm) were elevated with local anesthesia from the left lateral calf. Samples were immediately fixed in 4% paraformaldehyde and kept at 4°C for at least 2 days. The study was performed with the approval of the Institutional Ethics Committee of the Zhengzhou University (2015-81100949).

### Plasmid Constructs

Plasmids have been previously described (Wang et al., 2014). Briefly, the mammalian expression plasmid pKH3-HA-PINK1 was a kind gift from Dr. Bin Li (University of Science & Technology of China). Full-length BAG5 cDNA was amplified from a human fetal brain library (Invitrogen) using the primers 5′-cggaattctatgcgtttccattggttaccc-3′ and 5′-cgcggatccgtactcccattcatcaga-3′ and inserted into pcDNA3.1(+)/myc-HisA vector at *Bam*HI/*Eco*RI sites. pEGFP-BAG5 was constructed by subcloning a fragment excised from pcDNA3.1(+)/myc-HisA-BAG5 into pEGFP-N3 vector at *Bam*HI/*Eco*RI sites. All constructs were sequenced to confirm their fidelity. HA-PINK1^R492X^ was constructed similarly with HA-PINK1 (Che et al., 2013).

### Cell Culture, Transfections, and RNA Interference

Human embryonic kidney 293 (HEK293) cells (purchased from the Type Culture Collection of the Chinese Academy of Sciences, Shanghai, China) were maintained in DMEM (GIBCO, United States) supplemented with 10% newborn calf serum (GIBCO, United States), 100 U/ml penicillin and 100 mg/ml streptomycin (Invitrogen, United States), at 37°C in a humidified incubator of 5% CO_2_. The mammalian expression plasmid enhanced green fluorescent protein (EGFP)-BAG5, haemagglutinin (HA)-PINK1 and HA-PINK1^R492X^ were gifts provided by Professor Guanghui Wang (Che et al., 2013) (Soochow University, China). The HA tag was added to the N-terminal of PINK1 or PINK1 R492X, the expression was confirmed by immunostaining (Supplementary Figure 1). Transfections were performed using Lipofectamine 2000 (Invitrogen, United States) according to the instructions. Cycloheximide (CHX) was purchased from Sigma and carbobenzoxy-Leu-Leu-leucinal (MG132), a proteasome inhibitor, was obtained from Calbiochem. Plasmids and Lipofectamine 2000 were premixed in OPTI-medium (GIBCO, United States) for 30 min and then applied to the cells. After transfection for 6 h, the medium was replaced with fresh medium containing 15% FBS, and cells were treated for another 24 h and harvested subsequently. According to the manufacturer’s instructions (Invitrogen), 50 pmol of BAG5 siRNA was transfected using Oligofectamine. Oligo RNA was purchased from Gene-Pharma (Shanghai, China) containing the following sequences: siBAG5 sense, 5′-GGAGAUAUUCAGCAAGCUATT-3′, siBAG5 antisense, 5′-UAGCUUGCUGAAUAUCUCCTT-3′; siRNA control sense, 5′-UUCUCCGAACGUGUCACGUdTdT-3′, siRNA control antisense, 5′-ACGUGACACGUUCGGAGAAdTdT-3′.

### Immunoprecipitation and Western Blotting Analysis

Whole cell lysates were sonicated in lysis buffer [50 mM Tris–HCl (pH 7.5), 150 mM NaCl, 1 mM EDTA, 1 mg/ml aprotinin, 10 mg/ml of leupeptin, 0.5 mM Pefabloc SC, 10 mg/ml of pepstatin, 1% NP-40]. Cellular debris was removed by centrifugation at 12,000 × *g* for 20 min at 4°C. The supernatants were incubated with the antibodies in 0.01% BSA for 4 h at 4°C. After incubation, protein G Sepharose (Roche, Switzerland) was used for precipitation. The beads were washed with 1 × PBS for six times, and proteins were eluted with SDS sample buffer for immunoblot analysis. The samples were subjected to SDS-PAGE. After transferred to nitrocellulose membranes, blots were blocked with 15% non-fat dry milk in TBST (0.25% Triton X-100 in PBS, pH 7.4) for 1 h, and then incubated with primary antibodies overnight at 4°C. After washing three times in TBST, the membrane was incubated with anti-rabbit IgG (Cell Signaling Technology, United States) or anti-mouse IgG (Cell Signaling Technology, United States) for 1 h. Membranes were washed for three times and proteins were visualized using an ECL detection kit (Pierce Chemical, United States). The primary antibodies used were mouse monoclonal anti-HA tag antibody (Abcam, United Kingdom), rabbit polyclonal anti-HA tag antibody (Cell Signaling Technology, United States), rabbit polyclonal anti-GAPDH antibody (Cell Signaling Technology, United States), mouse monoclonal anti-BAG5 antibody (Abcam, United Kingdom), rabbit polyclonal anti-green fluorescent protein (GFP) antibody (Abcam, United Kingdom), and rabbit polyclonal anti-ubiquitin antibody (Cell Signaling Technology, United States).

### Immunohistochemistry and Immunohistofluorescence

HEK293 cells were washed with 1 × PBS and fixed with 4% paraformaldehyde for 5 min. Then cells were incubated with 0.25% Triton X-100 for 15 min and blocked in 4% FBS for 20 min, subsequently incubated with the primary antibodies overnight at 4°C. Frozen skin tissue sections were washed in 1 × PBS for five times and incubated with 0.3% Triton X-100 at room temperature for 30 min. After blocking the non-specific binding sites using 1 × PBS containing 2% BSA, the skin tissue sections were incubated with the primary antibodies overnight at 4°C. Following three washes with 1 × PBS containing 0.3% Triton-100, fluorescein isothiocyanate (FITC)-conjugated goat anti-mouse IgG was applied as secondary antibody (Vector Laboratories, United States) for 2 h at room temperature. Images were captured by using Nikon Labphoto-2 fluorescence microscope. The human subject studies were approved by the Institutional Ethics Committees of the Zhengzhou University. Written informed consent for skin biopsy was obtained from all patients and healthy individuals participating in the study.

### Statistical Analysis

All statistical analyses were performed using Student’s *t*-test or one-way ANOVA using SPSS statistical software package (SPSS version 8.0). Data were shown as mean ± SD or mean ± SEM. A *p* value less than 0.05 was considered statistically significant.

## Results

### Expression of BAG5 in the Skin Tissues From Patients With R492X *PINK1* Mutation Is Lower in Comparison to Health Controls

**FIGURE 1:**
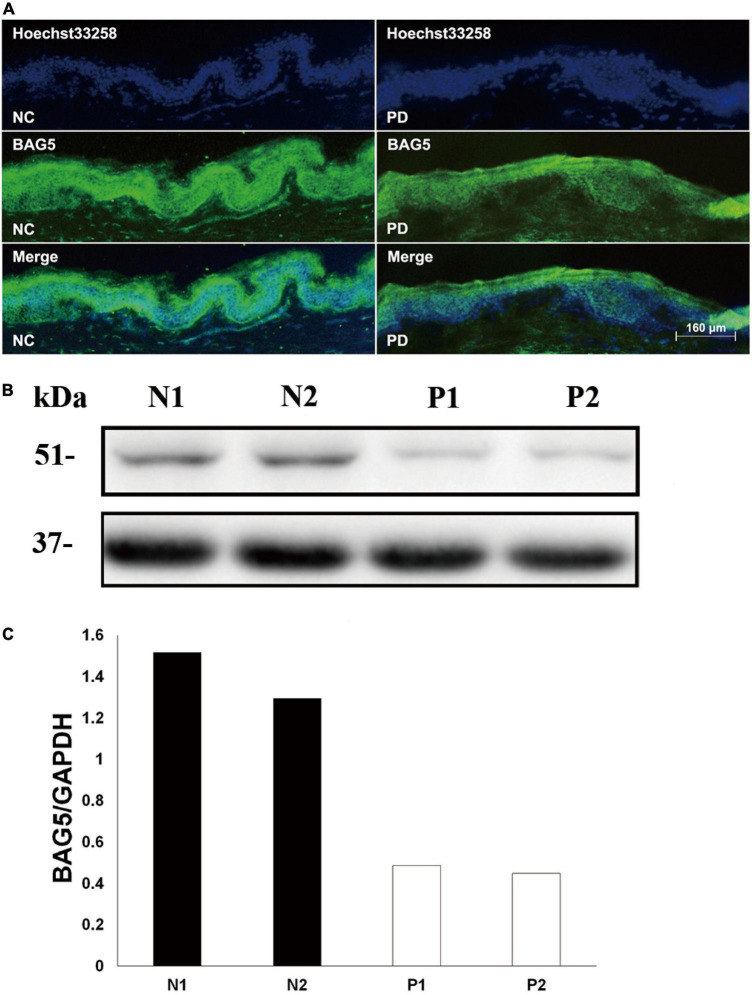
Decreased level of BAG5 in the skin tissue of patients with R492X *PINK1* mutation. **(A)** Immunofluorescence staining for the expression of BAG5 in skin tissues from healthy individuals (NC, left) and patients with PD harboring R492X *PINK1* mutation (PD, right). **(B)** Western blotting was performed using skin tissues from healthy individuals (N1 and N2) and PD patients with R492X *PINK1* mutation (P1 and P2). **(C)** Quantitative data from **(B)** (Density of endogenous BAG5 against GAPDH) using NIH ImageJ. The data represent the intensity of BAG5 band of the healthy individuals (N1 and N2) or PD patients with R492X *PINK1* mutation (P1 and P2), respectively. GAPDH serves as a loading control.

The BAG family proteins exhibit multiple functions including apoptosis and protein degradation (Bracher and Verghese, 2015), and previous studies have shown that BAG5 inhibited both Parkin E3 ligase and Hsp70 chaperone activities thereby enhancing dopaminergic neuron degeneration (De Snoo et al., 2019; Chen et al., 2020). We then hypothesized that BAG5 is involved in the pathogenesis of R492X *PINK1* mutation in autosomal-recessive PD. To test this hypothesis, we performed skin biopsy from two patients with PD harboring R492X *PINK1* mutation and two healthy controls. We found that the decreased level of BAG5 was detected in patients with R492X *PINK1* mutation compared with healthy control subjects by immunofluorescence (Figure 1A) and Western blotting analysis (Figures 1B,C). These results suggest that BAG5 is implicated in the pathogenesis of R492X *PINK1* mutation in PD.

### BAG5 Interacts With R492X Mutated PINK1 Protein

**FIGURE 2:**
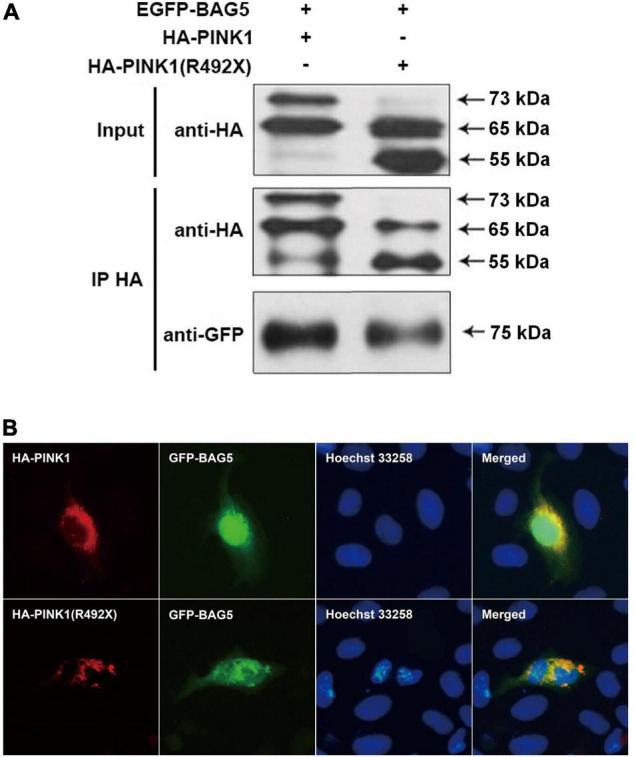
BAG5 interacts with R492X mutated PINK1 protein. **(A)** BAG5 interacts with PINK1 and R492X mutated PINK1 protein. We performed co-IP assays using HEK293 cells expressing GFP-BAG5 and HA-PINK1 or HA-PINK1^R492X^. Cell lysates were subjected to co-IP with rabbit polyclonal anti-HA tag antibody. The IP and input lysates were analyzed by immunoblotting with mouse monoclonal anti-HA tag antibodies. The results showed that BAG5 interacts with both wild type PINK1 and R492X mutated PINK1 protein. **(B)** Co-localization of GFP-BAG5 and HA-PINK1^R492X^. HEK293 cells were transfected with EGFP-BAG5 and HA-PINK1 or HA-PINK1^R492X^. Images show regional co-localization of BAG5 with R492X mutated PINK1 protein.

To determine the relationship between BAG5 and R492X mutated PINK1 protein, we first used co-immunoprecipitation (co-IP) experiments to examine if there is a direct interaction between BAG5 and R492X mutated PINK1 protein. We co-transfected with EGFP-BAG5 and HA-PINK1 or HA-PINK1^R492X^ vectors in HEK293 cells. After immunoprecipitation with a rabbit polyclonal anti-HA tag antibody, the immunoprecipitants were subjected to immunoblot analysis with a mouse monoclonal anti-GFP or anti-HA tag antibody. EGFP-BAG5 was co-precipitated with HA-PINK1^R492X^, suggesting that BAG5 physically binds to R492 mutated PINK1 protein. The results are shown in Figure 2A. Then, we detected subcellular co-localization of GFP-BAG5 with HA-*PINK1* and HA-*PINK1*^R492X^ in HEK293 cells co-transfected with HA-*PINK1* or HA-*PINK1*^R492X^ and EGFP-BAG5. As shown in Figure 2B, HA-*PINK1* was distributed most in the mitochondrial-rich perinuclear region (Matsuda et al., 2013; Liu et al., 2017), and GFP-BAG5 was evenly distributed throughout the whole cell. Furthermore, HA-*PINK1* and GFP-BAG5 were co-localized in perinuclear region. HA-*PINK1*^R492X^ was distributed unevenly around the nucleus, and largely co-localized with GFP-BAG5 (Figure 2B). These results confirmed that BAG5 interacts with R492X mutated *PINK1* protein.

### BAG5 Promotes Degradation of R492X Mutated PINK1 Protein

**FIGURE 3:**
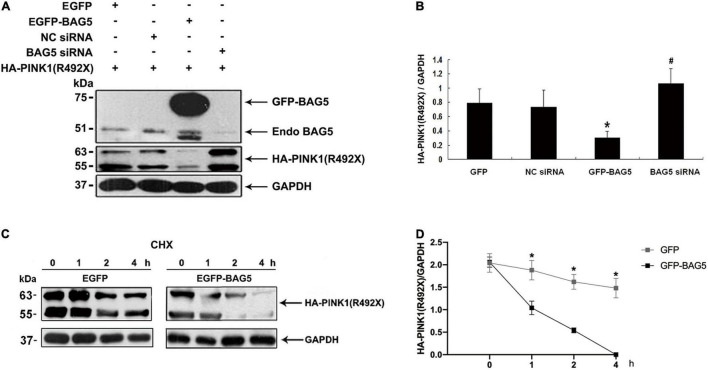
BAG5 promotes R492X mutated PINK1 protein degradation. **(A)** HEK293 cells were transfected with EGFP, EGFP-BAG5, negative control siRNA (NC siRNA), BAG5 siRNA and HA-PINK1^R492X^ for 36 h. Cells extracts were analyzed by immunoblot using the specific antibodies. HA-PINK1^R492X^ was decreased in GFP-BAG5-expressing cells, whereas knockdown of BAG5 increased the level of HA-PINK1^R492X^. **(B)** Quantitative data from A were shown. Values shown are the means ± SEM from the experiments. Level of statistical significance, *p < 0.05, ^#^*p* < 0.01. **(C)** HEK293 cells were transfected for 36 h with either EGFP or EGFP-BAG5 with HA-PINK^R492X^, then cells were incubated for the indicated times in the presence of 100 mg/ml CHX. Cells were resuspended in lysis buffer, and the proteins were analyzed by immunoblot using anti-HA tag antibody. **(D)** The expression level of HA-PINK1^R492X^ was significantly lower in GFP-BAG5-overexpressing cells than in GFP-overexpressing cells. Values shown are the means ± SEM, level of statistical significance, *p < 0.05. All experiments were performed more than thrice.

Previous studies showed that BAG5 was involved in regulating degradation of specific proteins by ubiquitin/proteasome-dependent pathways (Wang et al., 2014). We next investigated the effect of BAG5 on degradation of R492X mutated PINK1 protein. HEK293 cells were co-transfected with HA-PINK1^R492X^ and EGFP-BAG5 or EGFP-N1 (control) for about 36 h. The expression level of HA-PINK1^R492X^ was markedly lower in cells co-transfected with EGFP-BAG5, but not in cells co-transfected with EGFP tag alone (Figure 3A). To further confirm the role of BAG5 in degradation of R492X mutated PINK1 protein, we knocked down the expression of endogenous BAG5 by siRNA. As shown in Figures 3A,B, the down regulation of BAG5 significantly increases the expression of HA-PINK1^R492X^ in HEK 293 cells. Subsequently, we examined the stability of HA-PINK1^R492X^ in cells stably expressing either GFP or GFP-BAG5 after CHX treatment to block total protein synthesis. The poor expression of HA-PINK1^R492X^ in GFP-BAG5-overexpressing cells provides evidence that overexpression of GFP-BAG5 accelerates cellular degradation of HA-PINK1^R492X^ (Figures 3C,D).

### BAG5 Increases the Ubiquitination of R492X Mutated PINK1 Protein

**FIGURE 4:**
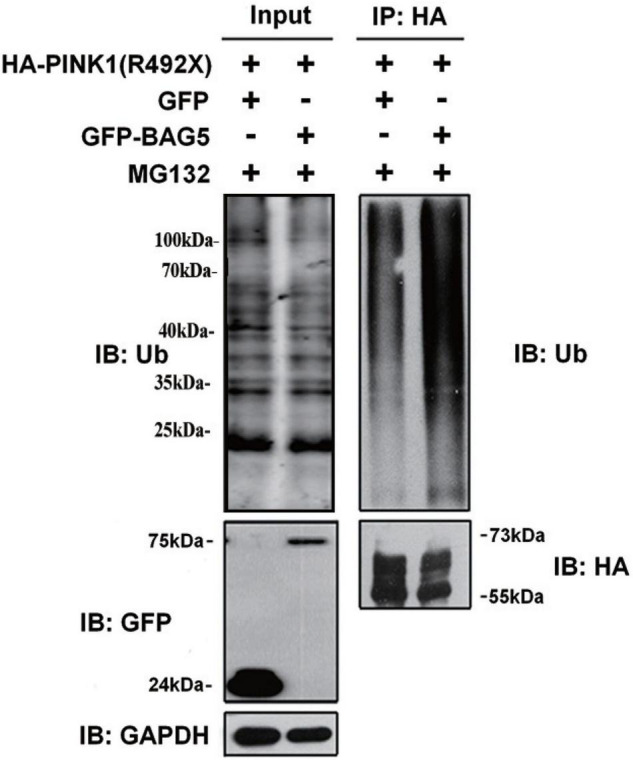
BAG5 increases ubiquitination of R492X mutated PINK1 protein. HEK293 cells were co-transfected with HA-PINK1^R492X^ and EGFP-N1 or EGFP-BAG5. The cells were treated with 10 mM MG132 for 12 h and subjected to immunoprecipitation using rabbit polyclonal antibodies against HA tag. Inputs and immunoprecipitants were subjected to immunoblot analysis using anti-ubiquitin, anti-EGFP, anti-HA tag or anti-GAPDH antibody.

Previous studies demonstrated that *PINK1* was mostly degraded by the ubiquitin-proteasome system (UPS) (Whiten et al., 2021). To explore whether overexpression of BAG5 increased the ubiquitination of R492X mutated *PINK1* protein, HEK293 cells were co-transfected with HA-PINK1^R492X^ and EGFP-BAG5 or EGFP-N1 for about 36 h, and then cells were treated with the proteasome inhibitor MG132 for 12 h. Cell lysates were collected and immuno-precipitated using affinity-purified rabbit anti-HA tag antibodies. As shown in Figure 4, overexpression of EGFP-BAG5 significantly increased the ubiquitination of HA-PINK1^R492X^ compared with EGFP control in the presence of MG132 (Figure 4). Thus, the results show that BAG5 accelerates the degradation of R492X mutated PINK1 protein via increasing ubiquitination of R492X mutated PINK1 protein.

## Discussion

In this study, we show that the expression level of BAG5 in R492X *PINK1* mutated PD patients is much lower than that in healthy controls. We then demonstrated that BAG5 promoted the degradation of R492X mutated *PINK1*. Moreover, we reveal that BAG5 functions through physically interacting with R492X mutated PINK1 protein. The evidence from this study indicates that BAG5 functions as a modulator controlling the expression level of R492X mutated PINK1 protein. Decreased expression of BAG5 promotes R492X mutated PINK1 protein accumulation and mitochondrial dysfunction, which potentially enhances the cytotoxic effect of R492X mutated PINK1 protein in patients with R492X PINK1 mutation. Although, the interaction between endogenous BAG5 and R492X mutated PINK1 and how BAG5 expression is regulated in PD patients with such mutation need to be further investigated, these data highlight the therapeutic targets for PD patients with R492X *PINK1* mutation.

Bcl-2 associated athanogene family consists of 6 protein members, characterized by the same BAG domain, and is found to participate in cell proliferation and survival, increasing stress tolerance, and cancer development (Pattingre and Turtoi, 2022). BAG5 is exceptional in this group of protein since it consists solely of the five BAG domains (Bracher and Verghese, 2015). Ying et al. (2013) showed that BAG5 reduced the degradation of PTEN and maintained its levels via an ubiquitination-dependent pathway. Che et al. (2015) demonstrated that BAG5 stabilized pathogenic ataxin3-80Q by inhibiting its ubiquitination as determined by western blotting and co-immunofluorescence experiments. Qin et al. (2017) revealed that BAG5 could decrease DJ-1’s stability and reduce its function on mitochondrial protection. These studies indicated that BAG5, as a key chaperone regulator of heat shock proteins, regulates ubiquitin-mediated degradation of many other proteins. The authenticity of our results is verified in line with the property above of BAG5. Wang et al. (2014) demonstrated that BAG5 inhibited PINK1 degradation through direct interaction with PINK1 through the UPS and BAG5 protected mitochondria against neurotoxin MPP^+^- and rotenone-induced oxidative stress. Here, we show that BAG5 promotes R492X mutated PINK1 protein degradation in this study. Mutations in *PINK1* gene are the second most common cause of autosomal recessive early-onset PD (Wang et al., 2018; Li et al., 2021). In 2004, R492X *PINK1* mutation was first identified in autosomal recessive early-onset parkinsonism (Hatano et al., 2004). Previous study by Yuan et al. (2010) revealed that stable expression of the R492X mutated PINK1 protein, unlike the wild-type PINK1 protein, causes mitochondrial cytochrome C release and cellular apoptosis. The R492X mutation seems to be a dominant-negative or gain-of-function dominant mutation that can induce cellular mitochondrial dysfunction and oxidative stress, especially with the environmental neurotoxin (MPP^+^). Therefore, the mechanism by which BAG5 exhibits an opposite effect on the degradation of the two proteins is not clear and remains to be further investigated. A simple explanation might be that BAG5 is involved in cellular mitochondrial and oxidative stress response/modulation and has multiple functions, one of which leads to a change in protein structure. Individual properties and physiological situation of the R492X mutated PINK1 protein might have been changed, which consequently resulted in different reactions to BAG5.

## Conclusion

In summary, we show that the expression levels of BAG5 decline in the skin tissues from patients with R492X *PINK1* mutation compared with control cases. Further, our results help demonstrate that BAG5 promotes the degradation of R492X mutated PINK1 protein via the UPS pathway *in vitro*. Although further studies on expanded patient samples from a large cohort and on why BAG5 level is decreased in PD patients with R492X *PINK1* mutation are needed, the level of BAG5 in the biopsied skin may be used for an indication of the patients’ condition or a diagnostic biomarker of familial juvenile parkinsonism. This study may also highlight potential therapeutic effect of targeted regulation of BAG5 for PD patients with R492X *PINK1* mutation.

## Data Availability Statement

The original contributions presented in the study are included in the article/Supplementary Material, further inquiries can be directed to the corresponding authors.

## Ethics Statement

The studies involving human participants were reviewed and approved by the Institutional Ethics Committee of the Zhengzhou University, Henan, China. The patients/participants provided their written informed consent to participate in this study.

## Author Contributions

XW and EW conceived the research design, carried out the experiments, analyzed the data, and wrote the first draft of this manuscript. YF and DQ analyzed the data, wrote, reviewed, and revised this manuscript. YC, HT, and HL provided administrative, technical, and material support. All authors read and approved the final manuscript.

## Conflict of Interest

The authors declare that the research was conducted in the absence of any commercial or financial relationships that could be construed as a potential conflict of interest.

## Publisher’s Note

All claims expressed in this article are solely those of the authors and do not necessarily represent those of their affiliated organizations, or those of the publisher, the editors and the reviewers. Any product that may be evaluated in this article, or claim that may be made by its manufacturer, is not guaranteed or endorsed by the publisher.

## Supplementary Material

Supplementary Figure 1 Expression of C-terminal and N-terminal HA tagged PINK1^R492X^. The HA tag was added to the C-terminal **(A)** or N-terminal **(B)** PINK1^R492X^, the expression was confirmed by immunostaining.

## Abbreviations

BAG5: Bcl2-associated athanogene 5
BD: BAG domain
CHX: cycloheximide
co-IP: co-immunoprecipitation
EGFP: enhanced green fluorescent protein
FITC: fluorescein isothiocyanate
GFP: green fluorescent protein
HA: haemagglutinin
MG132: carbobenzoxy-Leu-Leu-leucinal
MLS: mitochondrial localization signal
MTS: mitochondrial-targeting sequence
PD: Parkinson’s disease
*PINK1*: PTEN-induced kinase 1
*PINK1* R492X: PTEN-induced kinase 1 p.R492X mutation
PTEN: phosphatase and tensin homolog
TD: transmembrane domain
UPS: ubiquitin/proteasome system.
