# Supplementary figures and images for: Identification of BAG5 as a Potential Biomarker for Parkinson’s Disease Patients With R492X PINK1 Mutation
Source: Front Neurosci. 2022 Jul 27;16:903958. doi: 10.3389/fnins.2022.903958 (PMC9363588; doi:10.3389/fnins.2022.903958)

Supplemental Figure 1. Expression of C-terminal and N-terminal HA tagged PINK1<sup>R492X</sup>.

**A**

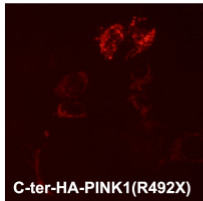

**B**

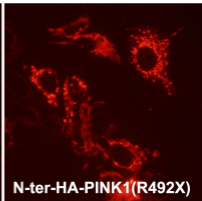

Supplement: Supplementary Figure 1 — Expression of C-terminal and N-terminal HA tagged PINK1R492X. The HA tag was added to the C-terminal (A) or N-terminal (B) PINK1R492X, the expression was confirmed by immunostaining. [file Image_1.pdf]
